# Structural characteristics of intestinal microbiota of domestic ducks with different body sizes

**DOI:** 10.1016/j.psj.2025.104930

**Published:** 2025-02-20

**Authors:** Hao Chen, Jiawei Li, Yongfei Wu, Yuhang Li, Sumei Zheng, Yan Wu, Rui Xuan, Liping Wu, Junjie Miao, Yanan Wang, Hongli Tan, Jing Zhou, Jianhua Huang, Xueming Yan

**Affiliations:** Key Laboratory of Natural Microbial Medicine Research of Jiangxi Province, College of Life Sciences, Jiangxi Science and Technology Normal University, Nanchang 330013, China

**Keywords:** Domestic duck, Gut Microbiome, Body size, 16S rRNA, Metagenome

## Abstract

Domestic ducks are economically important agricultural animals, and their body size is a crucial economic trait. The intestinal flora plays a pivotal role in influencing body metabolism, growth, and development. Currently, no literature is available on the potential effect of the intestinal flora of domestic ducks on body size. This study used 16S rRNA sequencing technology to investigate the fecal microbiota of 229 individuals reared under identical feeding conditions. The findings revealed that partridge ducks with large body sizes (LBS) exhibited a higher level of intestinal microbial diversity than ducks with small body sizes (SBS). Notably, the gut microbiota composition of SBS displayed significantly elevated proportions of *Streptococcus, Rothia*, and *Psychrobacter* compared to their counterparts with LBS. Conversely, *Lactobacillus* was significantly more abundant in LBS. *Jeotgalibaca* and *Psychrobacter* were identified as key biomarkers of SBS, whereas *Lactobacillus* and *Bacteroides* were predominant biomarkers of LBS. Functional predictions based on intestinal microbiota indicated discernible differences among different body types, particularly evident in non- partridge ducks. The present study investigated the correlation between the intestinal microbiota and body size of domestic ducks, aiming to provide practical insights for the production management of domestic duck farming.

## Introduction

Domestic ducks (commonly known as ducks) belong to *Anas platyrhynchos, Anas poecilorhyncha* and are domesticated from wild ducks (Hackett et al., 2008). As important agricultural and economic animals, domestic ducks are an important source of meat, eggs, and feather products (Huang et al., 2013). Duck meat is currently the second-largest in production among poultry products. Duck farming has gradually become an important pillar of China's animal husbandry industry. Simultaneously, China is responsible for nearly 79 % of the world's duck meat production (https://www.helgilibrary.com/charts/which-country-produces-the-most-duck-meat/) but constitutes approximately 3.7 % of global duck meat exports (https://www.tridge.com/intelligences/duck-meat/export). Many local duck breeds have emerged with differences in body size, feather color, and economic use owing to the diverse geographical, ecological, and climatic conditions in China, as well as directional selection. Chinese domestic ducks can be classified based on their body size. The body size of Chinese domestic ducks can be categorized as large or small, and their distinct gut microbiota may be a key factor in maintaining health and adapting to diverse environments.

Animals harbor many microbial communities inside and outside their bodies. Extensive research on intestinal microbiota has been conducted since the 20th century. Continuous advancements in sequencing technology have facilitated researchers’ gaining a deeper understanding of microbiomes. Numerous studies have shown that intestinal microbiota is closely related to animal health maintenance, nutrient acquisition, and behavior (Ezenwa et al., 2012; Kim et al., 2012; Qi et al., 2022). The composition of intestinal microbiota is affected by various factors such as diet (Zhou et al., 2022), environment (Yang et al., 2022) and antibiotics (Ramirez et al., 2020), thereby altering the physiological state of the host.

Recent studies have increasingly demonstrated the critical role of intestinal microbiota in regulating growth, metabolism, and energy homeostasis in livestock. In pigs, certain microbial taxa, such as Firmicutes and Bacteroidetes, have been identified as key modulators of nutrient digestion and absorption, directly influencing growth rate and final body weight (Quan et al., 2018; Xiao et al., 2016). Similarly, in cattle, the composition and diversity of the rumen microbiota, particularly the abundance of fiber-digesting bacteria, have been shown to correlate with feed conversion efficiency and weight gain, highlighting the microbiome's role as a metabolic regulator (Shabat et al., 2016).

Beyond livestock, the gut microbiota's impact on body size has been observed in various species, including mammals. Turnbaugh et al. observed that the ratio of Firmicutes/Bacteroidetes significantly increases in obese mice (Turnbaugh et al., 2006). Similar phenomena were also found in obese children (Akagbosu et al., 2022); however, other studies have reported opposite results (Schwiertz et al., 2010). Currently, the studies on the intestinal microbial community of domestic ducks mainly focus on the effects of environment, sex and other factors on the characteristics of certain duck breeds, and a few studies are available on the intestinal microbiota of multiple duck breeds (Tian et al., 2020; Zhu et al., 2020b). Exposing Shaoxing ducks to high temperatures results in augmented microbial populations within the gastrointestinal tract, accompanied by alterations in metabolic and transcriptional pathways (Tian et al., 2020).

Body size has significant research value as an important economic trait of animals, and is mainly determined by factors such as genetics, environment, and behavior. Currently, research on body size mainly focuses on the genetic level, and the influence of animal gut microbiota on body size remains unclear (Sun et al., 2019; Wu, et al., 2022). Therefore, investigations into the community structure and diversity of intestinal microbiota in domestic ducks of varying body sizes may yield valuable insights into their overall health, growth, and performance.

This study compared the composition and diversity of intestinal microbiota in indigenous Chinese duck breeds of different body sizes (large body size (LBS) and small body size (SBS)) using 16S rRNA sequencing and metagenomic sequencing technology. The functions of the microbiota were predicted based on the composition and abundance of genes in the metagenome. In order to further determine the effect of gut microbiota on body size, we also analyzed the gut microbiota of partridge and non-partridge ducks of different body sizes. The purpose of this study was to investigate the differences in the gut microbiota between large and small body size groups of domestic ducks to provide a reference for the protection and utilization of local duck breeds.

## Materials and method

### Sample collection

All the animals used in this study were obtained from the National Waterfowl Resource Library of Quanzhou City, Fujian Province. A total of 229 fecal samples were collected from 14 Chinese breeds of 80-day-old female ducks, and specific collection information is shown in Table 1. To minimize environmental influences on gut microbiota composition, all ducks were housed in separate groups based on breed, rather than in cages, allowing for natural social interactions while reducing stress-related microbiota variations. Experimental animals were provided with ad libitum access to food and water, ensuring that dietary intake was not a limiting factor. Additionally, the diet composition and water source were consistent across all groups to further control for potential environmental confounders. None of the individuals in this study were administered drugs such as antibiotics. The sampling equipment was disinfected with 75 % alcohol before the operation and each individual was separately sampled to reduce the risk of cross-infection. The fresh fecal samples were placed in sterile cryotubes and stored in liquid nitrogen. The samples were stored at -80°C until DNA extraction.

**Table 1:** Sample information.

Breed/Line	Average weight (kg)	Abbr.	Sample Type	Num. of Samples			
				16S	Total	Metagenome	Total
Jingding duck	1.796±0.098	JDD	Partridge duck	20	156	-	18
Chaohu duck	1.76±0.17	CHD		22		3	
Zhongshan Partridge duck	1.7	ZSP		13		3	
Shaoxing duck	1.547±0.192	SXD		19		-	
Sansui duck	1.497±0.186	SSD		15		-	
Shan Partridge duck	1.44±0.142	SPD		20		3	
Jinyun Partridge duck	1.3	JYP		20		3	
Mawang duck	1.258±0.143	MWD		17		3	
Youxian Partridge duck	1.223±0.05	YXP		10		3	
Longsheng Cui duck	1.806±0.137	LSD	No-Partridge duck	9	73	3	12
Ji’ an Red duck	1.795±0.137	JRF		20		3	
Liancheng White duck	1.487±0.115	LCW		16		3	
Brown tsaiya	1.456±0.157	TWD		11		-	
Putian Black duck	1.454±0.145	PTB		17		3	
Total	-	-	-	N = 229		N = 30	

### DNA extraction and 16S rRNA gene amplicon sequencing of fecal matter

We extracted genomic DNA of the microbial community from 229 fecal samples using the hexadecyltrimethylammonium bromide (CTAB)/ sodium dodecyl sulfate (SDS) method. The concentration and integrity of DNA were evaluated using 1 % agarose gel electrophoresis. Each qualified DNA sample was diluted to 50–100 ng/µL for the preparation of a 16S rDNA full-length library. The V4 hypervariable region of 16S rRNA was amplified using universal bacterial primers (515F: 5’-GTGCCGCCGCGGTAA-3’ and 806R: 5’-GGACTACHVGGGTWTCTAAT-3’) with indexes and adapters connected to the universal primers for polymerase chain reaction (PCR) amplification. The amplification was performed in a 10 µL reaction mixture containing 3 µM forward primer, 2 µM reverse primer, 2 ng extracted genomic DNA, and 15 µL 2 × Phanta Max super-fidelity DNA polymerase (New England Biolabs, USA). The cycling conditions were as follows: initial denaturation at 95 °C for 5 min, followed by 30 cycles of denaturation at 95 °C for 15 s, annealing at 50 °C for 30 s, extension at 72 °C for 30 s, and a final extension at 72 °C for 5 min. After amplification, the PCR products were extracted using a 1 % agarose gel and purified using a Qiagen Gel Extraction Kit (Qiagen, Germany) according to the manufacturer's instructions. Purified amplification products were quantified and normalized to prepare sequencing libraries. The quality of the sequencing library was evaluated using a Qubit 2.0 Fluorometer (Thermo Scientific, Waltham, MA, USA) and an Agilent Bioanalyzer System 2100. Subsequently, qualified libraries were sequenced on the Illumina NovaSeq platform.

### Analysis of 16S rRNA sequencing data

The original sequencing data were screened and processed according to the sequence quality for subsequent analysis. FLASH (V1.2.7) (Magoc and Salzberg, 2011) was used to merge paired-end reads, resulting in the generation of an original label. QIIME (V1.9.1) (Caporaso et al., 2010) was used to obtain high-quality clean tags. The UCHIME algorithm (Edgar et al., 2011) was employed to compare tags with a reference database (SILVA) to detect and remove chimeric sequences, resulting in high-quality sequencing data. Uparse software (V7.0.1001) (Edgar, 2013) was used to cluster valid sequences into the same operational classification unit (OTU) based on a 97 % similarity threshold. Based on the OTU results, the Naive Bayes model (NBC) algorithm (Wang et al., 2007) was used to annotate the OTUs sequences using SILVA as a reference database, with the species classification information corresponding to each OTU obtained. The abundance of OTUs was normalized using the sequence number standard corresponding to the sample with the lowest number of sequences.

The diversity of each sample was evaluated based on the abundance distribution of OTUs in the different samples. A relative abundance curve was used to evaluate the species richness and microbial diversity of each sample at different sequencing depths. Venn diagrams were drawn using the Venn Diagram package in R software to identify specific and shared OTU types among different samples. A number of α indexes (ACE and Simpson) were calculated to analyze α diversity among samples, and β diversity analysis was utilized to compare the similarity of community structure among different samples. We calculated four dissimilar matrices based on four commonly used distance algorithms. We selected the Weighted UniFrac distance that could explain the highest variation as the distance matrix for subsequent analysis owing to Due to the complex and diverse microbial communities in the environment. Principal coordinate analysis (PCoA) was employed at the OTU level to reveal differences in species complexity between different samples. The relative abundance of species in the samples was subjected to hierarchical clustering using R software, followed by correlation analysis and mapping. Furthermore, analysis of similarities (ANOSIM) was used to evaluate differences in the intestinal microbiota among the different groups.

We used the t-test in the STAMP software to check for significant differences at the genus level between the sample groups to analyze the differences in microbial communities between large and small body sizes. We applied linear discriminant analysis (LDA) effect size (LEfSe) to determine biomarkers between different body sizes, and exploited the Kruskal-Wallis test (α=0.05) and LDA score >3.5 as the threshold.

### Metagenomic sequencing and analysis

In this study, the metagenomic data were obtained through selective metagenomic sequencing based on the analysis results of 16S rRNA sequencing data. Three individuals from each breed within the large body size and small body size groups were selected for metagenomic sequencing, followed by metagenomic data analysis. We filtered 30 samples from different individuals for metagenomic sequencing analysis on the Illumina NovaSeq platform to further explore functional changes. The original data from the Illumina NovaSeq sequencing platform were processed using Readfq (V8, https://github.com/cjfields/Readfq) to obtain clean data for subsequent analysis. The specific processing steps were as follows: (a) deleting reads with low-quality bases (default quality threshold less than or equal to 38) above certain parts (default length is 40bp); (b) removing reads with N bases reaching a certain percentage (default length is 10bp); (c) deleting reads that overlap with Adapter on a certain part above (default length is 15bp). Soap Aligner software was used to filter out reads originating from the host (soap2.21, http://soap.genomics.org.cn/SoapAligner.html). High-quality reads of each sample were assembled using SOAPdenovo software (V2.04, http://soap.genomics.org.cn/SOAPdenovo.html) (Luo et al., 2012). After the independent de novo assembly of each sample, all unused reads in all samples were combined and mixed for assembly to maximize data utilization. Subsequently, we disconnected the assembled scaffolds from the N connection to obtain the scaftigs. Finally, further analysis was conducted using fragments exceeding 500bp in length from all scaftigs.

We used MetaGeneMark (V2.10, http://topaz.gatech.edu/GeneraMark/) to predict the ORFs of Scaftigs and mixed-assembly Scaftigs from each sample, ORFs < 100bp were filtered out. CD-HIT software (V4.5.8, http://www.biologistics.org/CD-HIT) was applied to reduce sequence redundancy for the predicted ORFs (Fu et al., 2012), and a unique initial gene catalog (where genes refer to nucleotide sequences that encode unique and continuous genes) was obtained (Sunagawa et al., 2015). SoapAligner (soap 2.21) was used to map clean data from each sample to a unique initial gene catalog to obtain the final gene catalog (Unigenes) for subsequent analysis. The abundance of each Unigene in each sample was statistically analyzed based on the number of mapped reads and gene length.

DIAMOND software (V0.7.9) was used to functionally annotate the metagenome, aligning single genes to the KEGG (version 201609, http://www.KEGG.jp/KEGG/) (Kanehisa et al., 2014), CAZy (version 20150704, http:-/www.CAZy.org/) (Cantarel et al., 2009), and Comprehensive Antibiotic Resistance Database (https://card.mcmaster.ca/) (Jia et al., 2017). The optimal alignment rate was employed for subsequent analysis of the alignment results of each sequence. The relative abundance at each functional level was equal to the sum of the relative abundances annotated at that functional level. Tax4Fun was used to infer the metabolic functions and pathways of the gut microbiota in Chinese ducks.

## Results

### Data overview

**Fig. 1:**
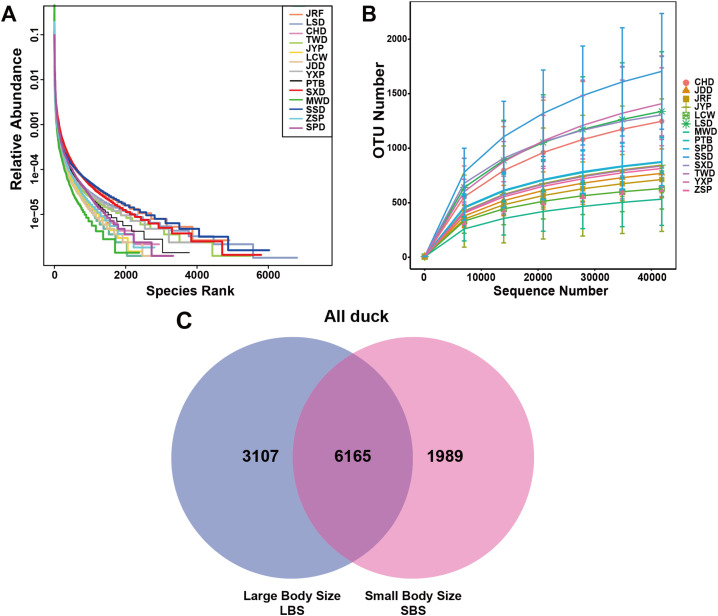
**Overview of sequencing data.** A. The relative abundance curve of all samples. B. Plot of OTU quantity for all varieties. C. The OTU Venn plots of different body types from all domestic ducks.

In this study, 88,570 raw tags were obtained from 229 duck samples. After a series of quality control and bioinformatics analyses, 86,142 clean tags were obtained. Our novel data-set consisted of 63,645 valid tags from these samples, with a quality control efficiency of 72.12 % and Q20 values > 99 % used for subsequent analysis (Table 2). We obtained a total of 14,575 OTUs following OTU clustering and annotation analysis. The relative abundance curve and OTU number of each breed showed a relatively flat curve as the number of sequences increased, indicating that sufficient reads were available to represent each microbial community and ensure the adequacy and accuracy of the analysis (Fig. 1A and B).

**Table 2:** Sequencing data statistics.

Sample Name	Raw PE (#)	Raw Tags (#)	Clean Tags (#)	Effective Tags (#)	Q20	Q30	GC%	Effective%
CHD	87526	85963	85311	64159	99.38	98.13	52.36	73.58
JDD	90062	87783	87021	64948	99.33	98.00	52.52	72.27
JRF	88332	86061	85371	63431	99.36	98.10	52.18	71.94
JYP	88374	87457	86906	64268	99.37	98.11	52.47	73.18
LCW	87794	86101	85541	65208	99.34	98.00	52.13	74.53
LSD	90091	88501	87843	65363	99.28	97.88	52.61	72.81
MWD	86647	85682	85225	65047	99.30	97.88	51.72	75.30
PTB	88179	86206	85714	59845	99.35	98.02	52.33	68.16
SPD	88240	85735	85043	63351	99.31	97.94	53.10	71.97
SSD	88345	87271	86731	62714	99.32	97.95	53.00	71.38
SXD	88980	85740	85087	61922	99.33	97.98	54.44	69.82
TWD	86382	84450	83900	63259	99.35	98.02	51.86	73.59
YXP	91582	90627	90012	63340	99.32	97.94	52.28	69.36
ZSP	91346	89629	89007	64773	99.35	98.07	52.46	71.06
Average	88570	86754	86142	63645	99.34	98.01	52.57	72.12

Note: Raw PE represents the PE reads of the original offline; Raw Tags refer to the tag sequence obtained by splicing; Clean Tags refers to the filtering of low quality and short length sequences by Tags; Effective Tags refer to the Tags sequence that is filtered for chimerism and ultimately used for subsequent analysis; Q20 and Q30 refer to the percentage of bases with mass values greater than 20 (sequencing error rate less than 1 %) and 30 (sequencing error rate less than 0.1 %) in Effective Tags; GC (%) represents the content of GC bases in Effective Tags; Effective (%) represents the percentage of the number of Effective Tags to the number of Raw PEs.

All experimental domestic ducks were divided into two populations: LBS and SBS. We selected four domestic duck breeds with the highest body weights and four with the lowest body weights as representative groups for comparative analysis of gut microbiota using 16S rRNA sequencing and metagenomics. Among them, the LBS consisted of LSD, JDD, JRF, and CHD, whereas the SBS consisted of SPD, JYP, MWD, and YXP. A total of 11,261 OTUs were obtained by 97 % similarity clustering of the annotated OTUs. Of these 6,165 were shared by LBS and SBS; 3,107 were unique OTUs for LBS; and 1,989 were specific to SBS (Fig. 1C). The body sizes of the partridge ducks were classified into two groups according to body size: CHD and ZSP for LBS, and MWD and YXP for SBS. A total of 4,206 shared OTUs were found among the different body sizes and the number of unique OTUs for LBS and SBS were 3,503 and 943, respectively (Fig.S1A). The non-Partridge ducks with LBS were LSD and JRF, whereas those with SBS were LCW and PTB. A total of 2,740 shared OTUs were found among the different body sizes with 2,922 and 1,871 unique OTUs to large non-partridge ducks and small non-partridge ducks, respectively (Fig. S1B). Among all body sizes, LBS had a higher number of OTUs than SBS, which may indicate that LBS has a more abundant gut microbiota.

### Diversity of intestinal microbial communities in ducks

**Fig. 2:**
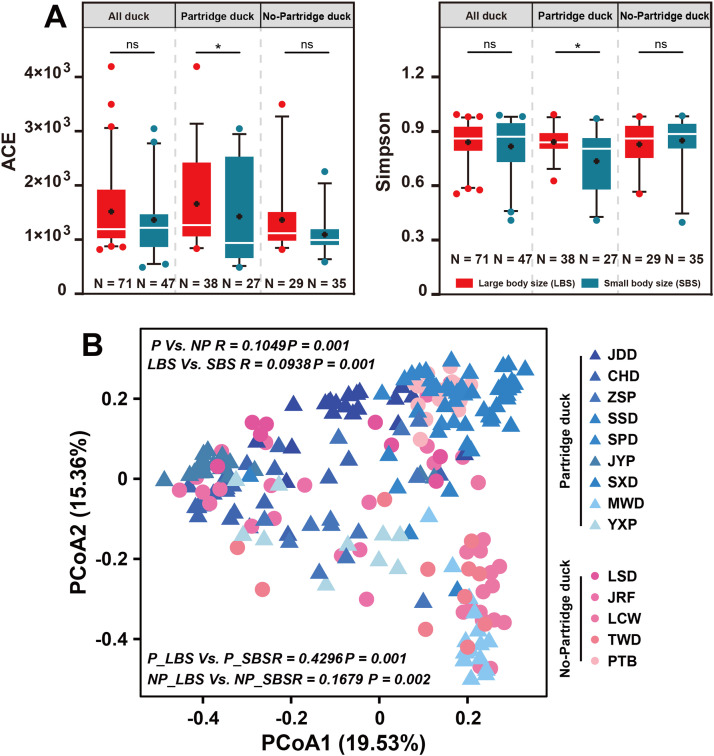
**Comparing gut microbial diversity in domestic ducks.** A. α index analysis between different groups (ACE index, Simpson index). B. Principal coordinate analysis (PCoA) diagram analysis and ANOSIM analysis between different groups.

We evaluated the diversity of the gut microbiota at the OTU level in LBS and SBS to verify these differences. Intestinal microflora richness and diversity were significantly different only in Partridge ducks of different body sizes, whereas no significant difference was observed in gut microflora richness and diversity among non-Partridge ducks of different body sizes according to alpha diversity indices (ACE and Simpson index) (Fig. 2A). Principal Coordinate Analysis (PCoA) was performed for each sample to verify whether any differences were present between the groups. Members with the same body size tended to cluster together individually, with no significant separation between the groups at first glance; however, significant differences persisted, indicating that they had different microbial communities. In addition, ANOSIM analysis further demonstrated significant differences in the microbial community structure between groups with different body sizes (Fig. 2B).

### Intestinal microbial community composition of ducks

**Fig. 3:**
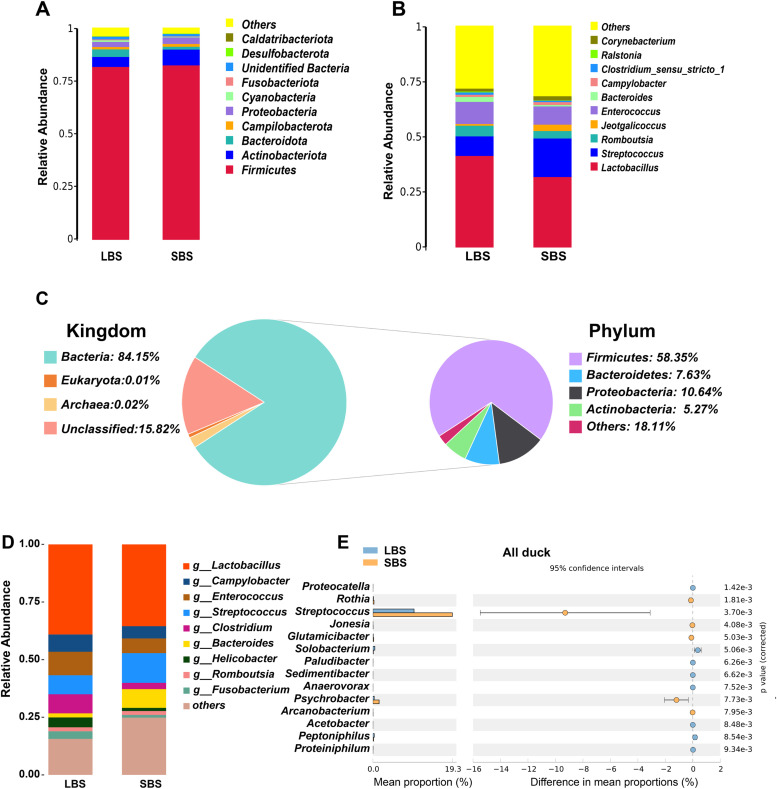
**Analyzing of phylum and genus level composition and different species of intestinal microorganisms in domestic ducks.** A. Horizontal composition of intestinal microbiomes in domestic ducks. B. Horizontal composition of intestinal microorganisms of domestic ducks. C. Taxonomic annotation of the gut gene catalog of domestic ducks at the metagenomic data Kingdom and Phylum level. D. Metagenomic data were used to determine the genus level composition of duck gut microbes. E Analysis of differences in genus levels of different body types in domestic ducks (top 15 genera with *P* < 0.01).

The gut microbial communities of domestic ducks mainly consisted of *Firmicutes, Actinobacteriota, Bacteroidota*, and *Proteobacteria* phyla, accounting for over 80 % of the total microbial community (Fig. 3A, Table S1). At the genus level, the intestinal microbiota of domestic ducks mainly comprised *Lactobacillus, Streptococcus, Romboutsia* and *Enterococcus* (Fig. 3B, Table S2). Furthermore, the metagenomic sequencing results of the gut microbes in domestic ducks showed that 84.15 % were annotated to the bacterial kingdom, and the main composition at the phylum level was consistent with the results of 16S rRNA data analysis (Fig. 3C). At the genus level, the annotation of the gut microbiome metagenomes of domestic ducks mainly included *Lactobacillus, Campylobacter, Enterococcus*, and *Streptococcus*, which differed from the results of the 16S rRNA data to some extent, possibly owing to the different numbers of samples (Fig. 3D). Simultaneously, the microbial community composition of different phyla and genera in partridge and non-partridge ducks was consistent with the gut microbial composition of all domestic ducks (Fig. S2, Table S3-S6).

Species differences at the phylum and genus levels were analyzed using MetaStat to further elucidate the differences in gut microbiota between different body sizes. Although no significant difference was found in the top 10 phylum-level analysis of abundance, Actinobacteriota and Proteobacteria had a higher proportion in SBS, while Bacteroidota had a higher proportion in LBS. Moreover, analysis of the top 15 species with significantly different abundances at the genus level showed that the proportions of *Rothia, Streptococcus, Jonesia, Glutamicibacter, Psychrobacter*, and *Arcanobacterium* in SBS were significantly higher than those in LBS (*P* < 0.01), while the abundance of *Proteocatella, Solobacterium, Paludibacter, Sedimentibacter, Anaerovorax, Acetobacter, Peptoniphilus*, and *Proteiniphilum* was significantly higher than those in SBS (*P* < 0.01) (Fig. 3E). Analysis of different species with different body sizes showed that *Streptococcus, Desemzia, Rothia*, and *Psychrobacter* were significantly more abundant in SBS, while the abundances of the other 11 genera were significantly higher in LBS (Fig. S3A). Moreover, differential species analysis of non- Partridge ducks showed that only *Lactobacillus* abundance was significantly higher in the LBS, while the proportion of other species was significantly higher in the SBS (Fig. S3B). In conclusion, *Streptococcus, Rothia*, and *Psychrobacter* were significantly higher in the gut microbiota of SBS compared with those in LBS, and the proportion of *Lactobacillus* in LBS was significantly higher.

### Identifying biomarkers of the intestinal microbiome in ducks

**Fig. 4:**
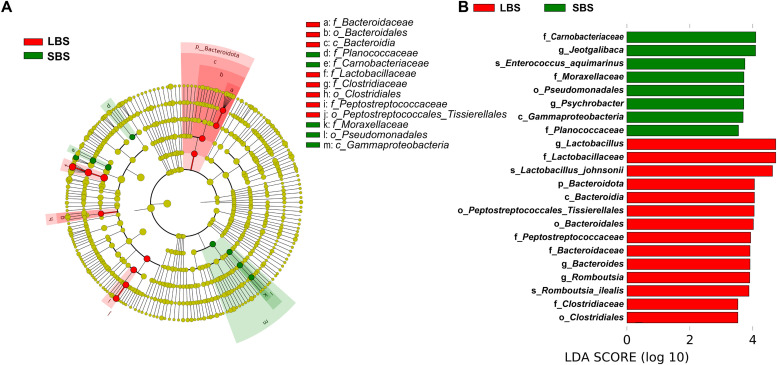
**LEfSe analysis of gut microbiota in different body sizes of domestic ducks.** A. Branching diagram representation of bacterial species with different abundance at different taxonomic levels. The roots of the branch plot represent the bounds, and the circles radiating from the inside out represent the taxonomic level from the gate to the species. The size of each node represents their relative abundance, with significantly different taxa painted in a specified color. B. Biomarkers with LDA> 3.5.

Linear discriminant analysis Effect Size (LEfSe) was used to identify biomarkers between LBS and SBS based on differential classification features. The branching diagram showed significant differences in bacterial species between the two groups at all levels. A total of 22 biomarkers were identified in all domestic ducks, with LDA score of >3.5 (Fig. 4). A total of 14 and 8 were biomarkers identified in LBS and SBS, respectively in all domestic ducks, among which Firmicutes and Bacteroides were dominant in LBS, while Firmicutes and Proteobacteria were dominant in SBS. Microorganisms at higher taxonomic levels were assumed to be unsuitable biomarkers; genus-level species are used as effective biomarkers. Two genus-level biomarkers were found in SBS (*Jeotgalibaca* and *Psychrobacter*), while three genera were found in LBS (*Lactobacillus, Bacteroides*, and *Romboutsia*).

A total of 20 biomarkers were identified among the Partridge ducks of different body sizes, of which 13 were identified in SBS. Three genus level biomarkers were identified: *Streptococcus, Jeotgalibaca*, and *Psychrobacter*. Seven biomarkers were identified for LBS, among which *Lactobacillus* was the only genus-level biomarker (Fig.S3A and B). Similarly, 21 biological markers were identified in non-partridge ducks. Among these, 12 biomarkers were identified in the SBS, and the genus-level biomarkers were *Enterococcus* and *Jeotgalibaca. Bacteroides* was the biomarker of the LBS (Fig.S3C and D). In summary, the biomarkers in SBS were mainly *Jeotgalibaca* and *Psychrobacter*, whereas those in LBS were mainly *Lactobacillus* and *Bacteroides*.

### Functional prediction of the Gut microbiome

**Fig. 5:**
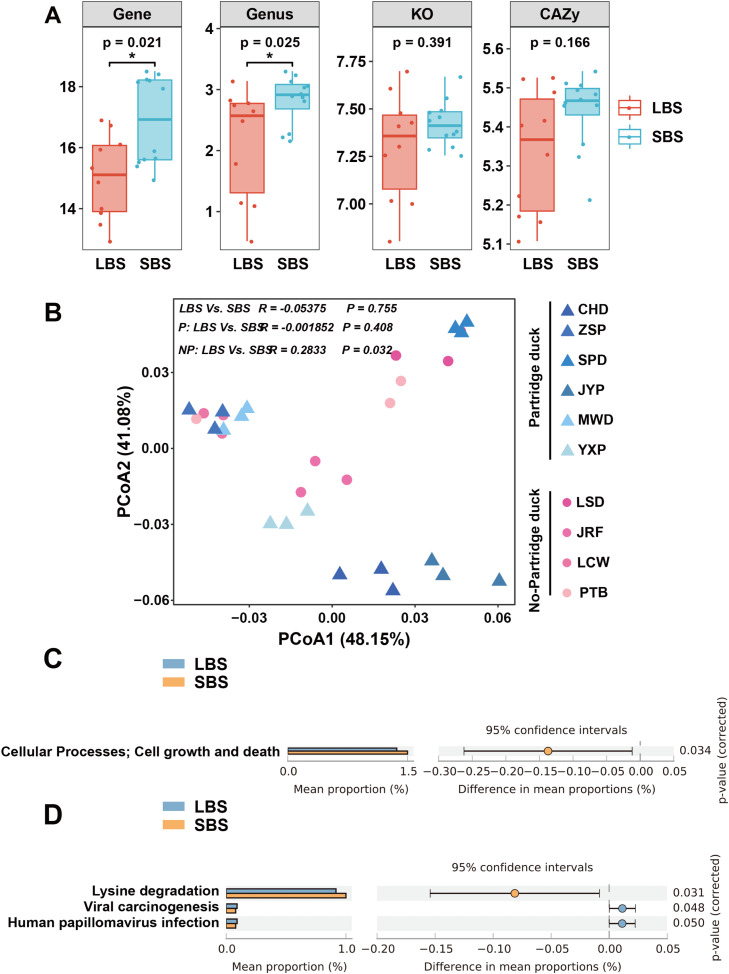
**Predicting intestinal microbial function in different body sizes of domestic ducks.** A. Microbial diversity at the gene, genus, KO, and CAZy levels (Shannon index). B. Functional PCoA analysis chart based on the KO level. C. Significant differences were observed in the prediction of KEGG secondary metabolic pathway function among all domestic ducks. D. Significant differences were threr in the prediction of KEGG tertiary metabolic pathway function among all domestic ducks.

The function of gut microbes in ducks of different body sizes was predicted using metagenomic data based on the relative abundance of genes, genera, KO and CAZy. The microbial diversity (Shannon index) of SBS was significantly higher than that of LBS in all domestic ducks of different body sizes (Fig. 5A). Functional PCoA analysis based on KO levels only revealed significant differences in non-partridge ducks, while functional differences were significant in all domestic ducks (Fig. 5B).

We performed functional predictions of Kyoto Encyclopedia of Gene and Genomes (KEGG) metabolic pathways to further analyze the functional differences in the gut microbiota among ducks of different body sizes. First, the functional prediction of the KEGG secondary metabolic pathway for domestic ducks of all body sizes revealed that only the growth and death of cells in SBS were significantly higher than those in LBS (Fig. 5C). Functional prediction of the KEGG tertiary metabolic pathway showed that lysine degradation was significantly increased in SBS, and viral carcinogenesis and human papillomavirus infection in LBS were significantly higher than those in SBS (Fig. 5D). The prediction of KEGG secondary metabolic pathways in partridge ducks showed that the functions of nucleotide metabolism and metabolism of other amino acids in LBS were significantly higher than those in SBS (Fig. S4A). Functional prediction of the KEGG tertiary metabolic pathway showed that the proportions of *Vibrio cholerae*, pyrimidine metabolism and cationic antimicrobial peptide (CAMP) resistance significantly increased in LBS and that cysteine and methionine metabolic, vitamin B6 metabolism and lipid acid metabolism in SBS were significantly higher than those in LBS (Fig. S4B). Only KEGG secondary metabolic pathway functions were predicted for non-partridge ducks owing to the significant differences in the functional PCoA mentioned above (Fig. S5). The gut microbiota of LBS was mainly involved in xenogenic biodegradation and metabolism, immune diseases, and membrane transport. Cell growth and death, glycan biosynthesis and metabolism, digestive system function, and transportation catabolism were significantly higher in SBS than in LBS.

## Discussion

Ducks play a pivotal role in China's poultry sector, with substantial economic importance. At present, China has a rich variety of local duck breeds with diverse characteristics that adapt to the local environment and exhibit different body sizes. The gut microbiota of ducks is closely related to host health, food digestion, nutrient absorption, and immune regulation (Naumova et al., 2021; Zhang et al., 2022). Some beneficial bacteria such as lactic acid bacteria can inhibit the growth of pathogenic bacteria, promote feed utilization efficiency, and improve the growth performance and immunity of ducks (Bai et al., 2023; Sun et al., 2022). In addition, the gut microbiota might be related to health problems such as intestinal diseases and an imbalance of gut microbiota in poultry (Chen et al., 2020). Although studies have been conducted on the diversity of gut microbiota in ducks classified by economic use (Ouyang et al., 2023), description of the effect of the gut microbiota on the body size of ducks is unavailable. Therefore, this study investigated the gut microbiota diversity of 14 indigenous duck breeds in China and analyzed the structural characteristics of the gut microbiota in ducks of different body sizes.

The α diversity of microbial communities is related to human health and the lower the α diversity, the worse the human health (Le Chatelier et al., 2013). In this study, we found that the α diversity of gut microbiota of LBS was significantly higher than that of SBS only among partridge ducks. This indicates that differences in the α-diversity of the gut microbiota may lead to variations in body size. While there was no significant difference in diversity among non-partridge ducks with different body sizes, this might be owing to the samples being in the same growth environment. At the phylum level, it was mainly composed of Firmicutes, Actinobacteriota, Bacteroidota, and Proteobacteria, similar to previous studies (Zhu et al., 2020a). A higher proportion of Actinobacteriota was present in SBS. Actinobacteriota is one of the most common bacterial phyla in the intestines of poultry. After infection, the relative abundance of *Actinobacteriota* in the gut microbiota of poultry significantly decreases (Kong et al., 2023; Li et al., 2023). The abundance of *Rothia* in *Actinobacteriota* was relatively high in SBS. *Rothia* can produce short-chain fatty acids (acetic acid, propionic acid, and butyric acid), decompose indigestible carbohydrates, and play an important role in the metabolism of important nutrients such as fat (Liu et al., 2022). Studies have shown that the number of *Rothia* in obese individuals has decreased, which might affect weight change in domestic ducks (Rosenbaum et al., 2015). *Psychrobacter* accounts for a high proportion of SBS as it is widely isolated from humans and patients with meningitis, making scientists suspect that it might be the cause of opportunistic infections in some patients (Maria et al., 2016; Welter et al., 2021). *Lactobacillus* has a large proportion in LBS compared with SBS. It is resistant to low pH and bile salts and plays an important role in maintaining a healthy intestinal environment (Krumbeck et al., 2016). In summary, changes in the gut microbiota may have an impact on the body size and intestinal health of domestic ducks.

Analysis of gut microbiota biomarkers in ducks of different body sizes indicated that differences in the gut microbiota affect their characteristics to some extent. *Jeotgalibaca* and *Psychrobacter* are the main representative biomarkers of SBS. *Jeotgalibaca* is positively correlated with the regulation of immune function in chickens and it is significantly associated with other immune diseases (Ni et al., 2020; Song et al., 2022). *Psychrobacter* may act as opportunistic pathogen, leading to animal diseases and impacting host health (Ortiz-Alcantara et al., 2016). Both biomarkers can potentially influence the overall well-being of the host, thereby affecting body size. *Lactobacillus* and *Bacteroides* were the main biomarkers of LBS. The probiotic *Lactobacillus* can enhance immunity, reduce symptoms of irritable bowel syndrome (IBS) and inflammatory bowel disease (IBD), improve intestinal health, increase nutrient utilization, and promote the metabolism of bile acids (BAs) and short-chain fatty acids (Huang et al., 2022; Liu et al., 2021; Najafi et al., 2022). *Bacteroides* help decompose food and produce the nutrients and energy needed by the body, participating in many important metabolic activities in the human colon, including carbohydrate fermentation, nitrogen-containing substance utilization, and the biological transformation of bile acids and other steroids (Le et al., 2022; Tamana et al., 2021). Furthermore, *Bacteroides* can provide a certain degree of intestinal protection to prevent invasion by invasive pathogens. These biomarkers may explain the larger size of ducks. Therefore, significant differences were present in the gut microbiota biomarkers of ducks with different body sizes that affected their body size and health to some extent.

Three KEGG tertiary metabolic pathways were identified with functional differences in the gut microbiota of all domestic ducks with different body types using Tax4Fun to infer the metabolic pathways and functions of the Chinese duck intestinal microbiota. Lysine degradation was higher in small ducks, and *Rothia* in the intestine can convert lysine into short-chain fatty acids, such as propionic and butyric acids, and decompose indigestible carbohydrates, which might be owing to the higher abundance of *Rothia* in SBS (Liu et al., 2022; Tain et al., 2023). Eight differences in KEGG tertiary metabolic pathway functions were identified among the different body types of partridge ducks. The metabolism of cysteine and methionine, vitamin B6, and fatty acids was significantly higher in the SBS group. This may be due to the higher abundance of *Actinobacteriota* in the SBS, which can degrade organic matter and produce various natural drugs, enzymes, and other substances (Ventura et al., 2007). Metabolic pathways of *Vibrio cholerae*, pyrimidine metabolism, and cationic antimicrobial peptide (CAMP) resistance were significantly associated with LBS. The growth of *Vibrio cholerae* can be suppressed by *Bacteroides vulgatus*, a prevalent biomarker in large ducks. However, the abundance of *Bacteroides* in the LBS did not necessarily inhibit the occurrence of *Vibrio cholerae* (You et al., 2019). In addition, the prediction of KEGG secondary metabolic pathway function in non-partridge ducks showed that the biosynthesis and metabolism of glycans, digestive system, transport and decomposition metabolism in SBS were significantly higher. Energy metabolism was related to low feed efficiency (Du et al., 2020), which may explain the small body size of ducks. Secondary metabolic pathways such as biological degradation and metabolism of heterologous organisms and membrane transport in LBS showed strong activity, which proved that the metabolism of LBS was more frequent, indicating that it maintained strong growth (Wang et al., 2022).

## Conclusion

This study analyzed the composition of the gut microbiota of local Chinese ducks of different body sizes and demonstrated significant differences in the composition, community structure, and function of the intestinal microbiota between large and small ducks. Only the α diversity of intestinal microbiota of LBS was significantly higher than that of SBS and LBS had a more complex community structure, which might improve the functional advantage of energy metabolism and bioavailability. Ducks of different body sizes showed significant differences in their intestinal microbiota biomarkers. The biomarkers for LBS were *Lactobacillus* and *Bacteroides*, which showed strong nutrient absorption, decomposition, and storage capabilities. Moreover, the analysis of metabolic pathway differences between LBS and SBS showed that the differences in functional metabolic pathways were more significant in non-partridge ducks.

## Ethics approval

The protocol of this experiment was approved by the Animal Ethics Supervisory Committee of Jiangxi Science and Technology Normal University (Y202334) and was performed in accordance with Animal Welfare China guidelines.

## Disclosures

The authors declare that they have no competing interests.

## Declaration of competing interest

The authors declare that they have no competing interests.

## Appendix Supplementary materials

Image, application 1

Image, application 2
