# Supplementary material for: Structural characteristics of intestinal microbiota of domestic ducks with different body sizes
Source: Poult Sci. 2025 Feb 20;104(4):104930. doi: 10.1016/j.psj.2025.104930 (PMC11930160; doi:10.1016/j.psj.2025.104930)
Supplement: Supplementary file 1 [file mmc1.docx]

**Supplementary Information**

**Additional file 1: Table S1** **Relative abundances of the top phyla at different body size.**

**Table S2 Genera of ducks with significant differences in gut microbes among different body sizes (P < 0.05).**

**Table S3 Relative abundances of the top 10 phyla at different body sizes.**

**Table S4 Genera of partridge ducks with significant differences in gut microbes among different body sizes (P < 0.05).**

**Table S5 The relative abundances of major phyla at different body size (abundance of top 10 phyla).**

**Table S6 Genera of non-partridge ducks with significant differences in gut microbes among different body sizes (P < 0.05).**

**Additional file 2:**

**Fig. S1 Venn plot of OTU levels in partridge ducks and non-partridge ducks.** A. Venn diagram of different body sizes of partridge ducks. B. Venn diagram of non-partridge ducks of different body sizes.

**Fig. S2 Histogram of species abundance for partridge ducks and non-partridge ducks.** A and C. Histogram of species of the top 10 most abundant phyla in partridge ducks (A) and non-partridge ducks (C). B and D. Histogram of species of the top 10 most abundant genera in partridge ducks (B) and non-partridge ducks (D).

**Fig. S3** **Analyzing genus level differences between partridge and non-partridge ducks.** A. Analysis of genus level body size differences in partridge ducks (the first 15 genera with *P* < 0.01). B. Analysis of genus level body size differences in non-partridge ducks (the first 15 genera with *P* < 0.01).

**Fig. S4 LEfSe analysis of gut microbiota in partridge and non-partridge ducks.** A. Branching map of bacterial species in different taxonomic levels of partridge ducks. B. Biomarkers of LDA> 3.5 in partridge ducks. C. Branching map of bacterial species in different taxonomic levels in non-partridge ducks. D. Biomarkers of LDA> 3.5 in non-partridge ducks.

**Fig. S5 Predicting gut microbiota function in different body sizes of partridge ducks.** A. Predicting KEGG secondary metabolic pathway function in partridge ducks. B. Predicting KEGG tertiary metabolic pathway function in partridge ducks.

**Fig. S6 Predicting KEGG secondary metabolic pathway function in non-partridge ducks.**
